# A conserved C-terminal peptide of sorghum phosphoenolpyruvate carboxylase promotes its proteolysis, which is prevented by Glc-6P or the phosphorylation state of the enzyme

**DOI:** 10.1007/s00425-021-03692-3

**Published:** 2021-08-05

**Authors:** Jacinto Gandullo, Rosario Álvarez, Ana-Belén Feria, José-Antonio Monreal, Isabel Díaz, Jean Vidal, Cristina Echevarría

**Affiliations:** 1 Departamento de Biología Vegetal, Facultad de Biología, Universidad de Sevilla, Avenida Reina Mercedes nº 6, 41012 Seville, Spain; 2 Centro de Biotecnología y Genómica de Plantas, Universidad Politécnica de Madrid, Campus de Montegancedo, Autovía M40 (km 38), Pozuelo de Alarcón, 28034 Madrid, Spain; 3 Institut de Biotechnologie des Plantes, UMR8618, Bâtiment 630, Université de Paris-Sud 11, 91405 Orsay, Cedex, France

**Keywords:** C_4_ photosynthesis, C19 peptide, C-terminus, Proteolysis regulation, Sorghum leaves

## Abstract

**Main conclusion:**

A synthetic peptide from the C-terminal end of C_4_-phosphoenolpyruvate carboxylase is implicated in the proteolysis of the enzyme, and Glc-6P or phosphorylation of the enzyme modulate this effect.

**Abstract:**

Phosphoenolpyruvate carboxylase (PEPC) is a cytosolic, homotetrameric enzyme that performs a variety of functions in plants. Among them, it is primarily responsible for CO_2_ fixation in the C_4_ photosynthesis pathway (C_4_-PEPC). Here we show that proteolysis of C_4_-PEPC by cathepsin proteases present in a semi-purified PEPC fraction was enhanced by the presence of a synthetic peptide containing the last 19 amino acids from the C-terminal end of the PEPC subunit (pC19). Threonine (Thr)944 and Thr948 in the peptide are important requirements for the pC19 effect. C_4_-PEPC proteolysis in the presence of pC19 was prevented by the PEPC allosteric effector glucose 6-phosphate (Glc-6P) and by phosphorylation of the enzyme. The role of these elements in the regulation of PEPC proteolysis is discussed in relation to the physiological context.

**Supplementary Information:**

The online version contains supplementary material available at 10.1007/s00425-021-03692-3.

## Introduction

Phosphoenolpyruvate carboxylase (PEPC; EC 4.1.1.31) catalyzes the addition of bicarbonate to phosphoenolpyruvate (PEP) to form oxaloacetate, which is reduced to malate by the enzyme malate dehydrogenase (MDH). The family of plant-type PEPCs (PTPCs) in sorghum includes four C_3_-type PEPCs and the photosynthetic C_4_-PEPC (Paterson et al. 2009). C_4_-PEPC catalyzes the first carboxylation step in C_4_ photosynthesis, and gene expression is activated during the greening of the C_4_ leaf when PEPC accumulates in the cytosol of mesophyll cells as required for the functioning of the C_4_ pathway (Chollet et al. 1996). C_4_-PEPC has been further studied in relation to its catalytic and regulatory properties and the biochemical and signaling mechanisms that control its subcellular activity (Chollet et al. 1996; Echevarría and Vidal 2003; Izui et al. 2004). C_3_-PEPCs are also key enzymes in the metabolism of carbon and nitrogen, with central roles in respiration, amino acid synthesis, and the development and germination of seeds (Chollet et al. 1996; O’Leary et al. 2011). All PTPCs have a conserved N-terminal seryl residue that is phosphorylated by PEPC kinases (PEPCks; Echevarría and Vidal 2003), whereas this residue is absent from bacterial-type PEPC (BTPC; Sánchez and Cejudo 2003). Phosphorylation involves an N-terminal regulatory serine (Ser8 in C_4_-PEPC from sorghum), and this regulatory post-translational modification (PTM) interacts with metabolite effectors to decrease its sensitivity to feedback inhibition by L-malate and increase its affinity for the allosteric activator Glc-6P and its Vmax (Echevarría and Vidal 2003; Nimmo 2003). PEPC is also subjected to different PTMs, such as monoubiquitination (Uhrig et al. 2008; Ruíz-Ballesta et al. 2014, 2016), nitric oxide-related PTMs (S-nitrosylation, Tyr-nitration), and PTMs associated with oxidative stress (carbonylation; Arias-Baldrich et al. 2017; Baena et al. 2017). Monoubiquitination of *Arabidopsis* C_3_-PEPC is selectively degraded by autophagy (Baena et al. 2021). However, it is not known whether C_4_-PEPC is modified by monoubiquitination, and evidence of a possible degradation mechanism controlling the amount of C_4_-PEPC in the cytosol of mesophyll cells is lacking.

The C-terminal 19 amino acid domain (hereafter, “C-term”) is highly conserved in all PTPCs sequenced thus far and is also conserved in prokaryotic PEPCs (Lepiniec et al. 1993; Chollet et al. 1996; Gehrig et al. 1998). It has been implicated in the stability of the protein (Grisvard et al. 1998; Dong et al. 1999), the maintenance of catalytic activity, and the oligomeric state of the enzyme (Dong et al. 1999). Limited 3′ deletion in the *Ppc* gene from *Escherichia coli* resulted in a decreased amount of the enzyme and suppression of its catalytic activity (Sabe et al. 1984). Studies of the recombinant C-terminal truncated C_4_-PEPC concluded that the conserved C-terminal QNTG tetrapeptide of sorghum C_4_-PEPC is indispensable for maximal catalytic activity but not for homotetramer formation (Dong et al. 1999). Crystallographic studies have deciphered the three-dimensional structure of the *E. coli* and maize (C_4_) PEPCs, making clear the contribution of the enzyme C-terminal end to active and inhibitor sites (Kai et al. 1999; Matsumura et al. 2002).

The C-term is embedded in a hydrophobic region of the protein subunit (Matsumura et al. 2002), and in sorghum the C_4_-PEPC enzyme can be found in vitro in two different conformational states that differ in the accessibility of their C-terminal tail to specifically designed antibodies (Álvarez et al. 2003). We have also previously reported that a synthetic peptide containing the last 19 amino acids of the C-term of C_4_-PEPC sorghum leaves (pC19) specifically inhibits the in vitro phosphorylation of enzymes by PEPCk (Álvarez et al. 2003).

Protein purification from dark-adapted sorghum leaves on hydroxyapatite and anion exchange on mono Q led to fractions highly enriched in C_4_-PEPC (sp-PEPC). Among the contaminating proteins in these fractions we have been able to detect the presence of cathepsin proteases, mainly of the B and L types (Gandullo et al. 2019; Plaxton 2019). We have also shown that PEPC proteolysis by these cathepsin proteases is highly increased by the presence of the anionic phospholipids phosphatidic acid (PA), phosphatidylinositol (PI), and lyso-phosphatidic acid (LPA), which in turn inactivates the enzyme by means of a conformational change clearly detected by the exposing of the C-term usually embedded in the native, active enzyme. Conversely, the allosteric activator Glc-6P returns the exposed C-term of PEPC to the embedded conformation (Gandullo et al. 2019), increasing the stability of the protein. These changes in the conformational state of the enzyme seem to be essential to its stability, as the exposed C-term conformation is highly sensitive to proteolysis by cathepsin proteases that co-purify with PEPC.

The fact that this sequence is highly conserved, and that it is modulated by regulators such as anionic phospholipids or Glc-6P (Gandullo et al. 2019), suggests a possible role for C-term in modulating PEPC activity, stability, or the aggregation state of the enzyme. Whether the mechanism based on the C-term PEPC has physiological significance in the C_4_ leaf is an open question in our group. Here we report that the addition of a synthetic C-term (19 amino acid) peptide to the semi-purified PEPC fraction (sp-PEPC) results in a rapid decrease in enzyme activity due to cathepsin-based degradation. This is modulated by the antagonistic effect of the activator Glc-6P and the extent of phosphorylation (Ser8 of the opposite N-terminal) of the enzyme subunit. A possible implication of these findings for the physiological mechanism that regulates PEPC degradation/amount in the cytosol of mesophyll cells is discussed.

## Materials and methods

### Materials

The peptides C19 ([Y]942EDTLILTMKGIAAGMQNTG960), C15 (C19 lacking the QNTG motif), Thr/Tyr-mutated C19 ([Y]942EDY_944_ LILY_948_MKGIAAGMQNY_959_G960), N-terminal dephosphorylated peptide N-t-OH (1-MASERHHSIDAQLRALAP-18), and N-terminal phosphorylated peptide N-t-OP (1-MASERHHS[PO_3_H_2_]IDAQLRALAP-18) were used in this study. The peptides LI (108LAHRRRNSKLKHGDFSDEGS127), L2 (333AEEVQSTPASKKVTKYYIEFWKQIPPNE360), and L3 (907SFKVTPQPPLSKEFADENKPAGLVKLN933), correspond to additional loops present in the computerized model of the sorghum C_4_-PEPC compared to the three-dimensional structure of *E. coli* PEPC. Peptides L2 and L3 were as hydrophobic as peptide C19. The L1, L2, and L3 peptides were supplied by PolyPeptide Group (Strasbourg, France). C19, C15, L1, L2, and L3 have been described previously (Álvarez et al. 2003). Specific polyclonal antibodies against the N-terminal peptide (N-t-IgGs) were raised against the N-t-OH peptide of sorghum C_4_-PEPC containing the phosphorylation motif (Pacquit et al. 1995). PEPC antibodies against C_4_-PEPC from sorghum leaves were prepared by the General Services of Research from Seville University. N-t-IgGs were supplied by PolyPeptide Group.

### Plant growth conditions

Sorghum plants (*Sorghum bicolor* [L.] Moench, var. PR87G57; Pioneer Hi-Bred, Madrid, Spain) were grown under controlled environmental conditions in a greenhouse under a 12 h photoperiod (350 µmol m^−2^ s^−1^, photosynthetically active radiation), a temperature of 28/20 °C (light/dark), and 60% relative humidity in hydroponic cultures with nitrate-type nutrient solution (Hewit 1966).

### Preparation of the semi-purified C_4_-PEPC fraction

All procedures were performed at 4 °C. Dark-adapted (12 h) sorghum leaves (20 g) were homogenized in a Waring blender with 100 mL extraction buffer containing 100 mM Tris–HCl pH 7.5, 5% (v/v) glycerol, 1 mM EDTA, 10 mM MgCl_2_, 14 mM β-mercaptoethanol, 1 mM phenylmethylsulfonyl fluoride (PMSF), 10 µg mL^−1^ chymostatin, 10 µg mL^−1^ leupeptin, 10 mM potassium fluoride, and 2% (w/v) polyvinylpyrrolidone (PVP). The homogenate was filtered through two layers of 80-µm nylon net and centrifuged at 45,000 g for 10 min. Proteins in the supernatant were precipitated by polyethylene glycol 8000 (PEG; 8.5–15%), then sedimented by centrifugation (45,000 g, 10 min). The pellet was dissolved in 7 mL buffer A containing 50 mM Hepes/KOH pH 7.1, 5 mM MgCl_2_, 1 mM EDTA, and 5 mM dithiothreitol (DTT). Hydroxyapatite (5 mL; Econo-Pac CHT-II, Bio-Rad, equilibrated with buffer A and Mono Q [5 mL]; catalog no. 723–4122, Bio-Rad Laboratories) chromatography was performed according to the procedure of McNaughton et al. (1989), except that the chromatography was done in a Bio-Rad Econo-System at low pressure. The final specific activity for sp-PEPC was 78.5 ± 5 U mg prot^−1^.

### Preparation of standard crude extract from leaves

The protein extracts used in Fig. 6 were obtained by grinding 0.2 g fresh weight (FW) leaf tissue using sand and 1 mL extraction buffer containing 100 mM Tris–HCl pH 7.5, 20% (v/v) glycerol, 1 mM EDTA, 10 mM MgCl_2_, 14 mM β-mercaptoethanol, 1 mM PMSF, and 10 µg mL^−1^ leupeptin. The homogenate was centrifuged at 12,000 g for 2 min to disrupt the mesophyll and bundle-sheath cells. The crude extract typically contained 6 U PEPC/mg protein. When indicated, sorghum leaves were illuminated at 700 µmol m^−2^ s^−1^ for 2 h before protein extraction.

### Proteolytic assay: standard and incubation with different peptides and metabolites

In most experiments, an aliquot of sp-PEPC (0.3 U) containing proteases, mainly cathepsin B and L (Gandullo et al. 2019), was incubated at 30 °C for 3 h with and without various synthetic peptides (C19, C15, Thr/Tyr-mutated C19, L1, L2, and L3) and metabolites (Glc-6P, PEP, or L-malate) in 50 µL of a medium containing 100 mM Tris–HCl pH 8, 20% glycerol, 5 mM MgCl_2_, 1 mM EDTA, 1 mM PMSF, and 10 µg/mL leupeptin. At the indicated time, aliquots (5 µL) were taken to measure PEPC activity at pH 8.0 and 2.5 mM PEP. Activity was expressed as a percentage of the initial activity. At the end of the incubation period, samples were analyzed by SDS–PAGE (10% [w/v] acrylamide) or native PAGE (7% [w/v] acrylamide) according to Laemmli (1970). All gels were stained with Coomassie Blue.

### Proteolytic assay using substrates containing AMC fluorophore

Hydrolysis of commercial substrates containing AMC (7-amino-4-methyl coumarin) fluorophore was performed after digestion of PEPC present in the sp-PEPC fraction overnight at 30 °C in the presence of pC19 (60 nmol). After this, commercial substrates were added. The proteolytic assay was performed on microplates. The optimal pH for each type of protease was used. The standard assay volume was 100 µL containing 25 µL sp-PEPC, and the corresponding substrate was added to a final concentration of 0.2 mM (Carrillo et al. 2011). Cathepsin B-like (Cat-B) and L-like (Cat-L) activity was assayed with Z-RR-AMC (N-carbobenzoxyloxy-Arg-Arg-7-amido-4-methylcoumarin) or Z-FR-AMC (N-carbobenzoxyloxy-Phe-Arg-7-amido-4-methylcoumarin) substrates, respectively, and a buffer containing 0.1 M citrate pH 6, 0.15 M NaCl, and 5 mM MgCl_2_ (Carrillo et al. 2011). The mixture was incubated at 37 °C for 24 h, and the emitted fluorescence was measured with a 365 nm excitation wavelength filter and a 465 nm emission wavelength filter. Blanks were used to account for the spontaneous breakdown of substrates, and results were expressed as µmol min^−1^ mL^−1^. The system was calibrated with a known amount of AMC in a standard reaction mixture.

### Assay of PEPC activity

PEPC activity was measured spectrophotometrically at an optimal pH of 8.0 using the NAD-dependent malate dehydrogenase (NAD-MDH)-coupled assay at 2.5 mM PEP (Echevarría et al. 1994). Assays were initiated with the addition of an aliquot of the enzyme preparation or crude extract. A unit of enzyme was defined as the amount of PEPC that catalyzed the carboxylation of 1 µmol PEP min^−1^ at pH 8 and 30 °C.

### *In vitro* phosphorylation with PKA and PEPC phosphorylation state

Aliquots of sp-PEPC (0.1 U PEPC) were phosphorylated in vitro at pH 8 using the catalytic subunit of cAMP-dependent protein kinase (PKA) from bovine heart (5 U) according to Echevarría et al. (1994). The phosphorylation state of PEPC was determined via the malate test (malate inhibition at a suboptimal pH of 7.3) and expressed as the IC_50_. A high IC_50_ is correlated with a high degree of PEPC phosphorylation (Echevarría et al. 1994).

### Electrophoresis and Western blotting

The samples were subjected to SDS–PAGE (10% [w/v] acrylamide) according to Laemmli’s (1970) method for 2 h at 100 V and room temperature in a Mini-Protean^®^ III-2D cell (Bio-Rad). For native PAGE, the samples were immediately mixed with sample buffer (Tris–HCl 100 mM, 20% [v/v] glycerol, and 0.05% [w/v] bromophenol blue) and analyzed by PAGE (7% [w/v] acrylamide). The final concentrations in the separation gel were as follows: Tris–HCL 375 mM pH 8.8, APS (0.5% [w/v]), and 6 mM TEMED. The stacking gel (4% [w/v] acrylamide) contained 125 mM Tris–HCl, pH 6.8, and was polymerized like the separating gel. The electrode buffer (pH 8.3) contained 0.025 M Tris–HCl and 0.192 M glycine. Electrophoresis was performed at 100 V for 3 h and at 4 °C with a Mini-Protean^®^ III-2D cell. After electrophoresis, proteins on the gels were stained with Coomassie Blue R-250 or electro-blotted onto a nitrocellulose membrane (N-8017; Sigma) at 10 V (5.5 mA cm^−2^) for 30 min in a semi-dry transfer blotting apparatus (Bio-Rad). Membranes were blocked in Tris-buffered saline (20 mm Tris–HCl and 0.15 mM NaCl pH 7.5) containing 5% (w/v) powdered milk. PEPC bands were immunochemically labeled by overnight incubation of the membranes at room temperature in 20 mL Tris-buffered saline containing 40 µg PEPC-IgGs (antibodies against C_4_-PEPC from sorghum leaves). Subsequent detection was performed with affinity-purified goat anti-rabbit IgGs and a chemiluminescence detection system (Super Signal West Dura Signal, Pierce) according to the manufacturer’s instructions. Native C_4_-PEPC has been described as a tetrameric enzyme with a molecular weight of 440 kDa (Chollet et al. 1996).

### Separation of C_4_-PEPC and C19 protease by native PAGE

All steps were performed at 4 °C. sp-PEPC (50 U) was analyzed by native PAGE (7% [w/v] acrylamide) as described above but in the absence of the stacking gel. After electrophoresis, we located the PEPC band by incubating the gel for 5 min in a medium containing the assay components of PEPC activity and 0.16 mM CaCl_2_. Pi released by the PEPC reaction precipitated as a white calcium phosphate (Ca_3_[PO_4_]_2_) band. The PEPC band was excised from the gel and the proteins electro-eluted for 12 h at 5 V (model 422 electro-eluter; Bio-Rad). The electro-eluted fraction was recovered (400 µL/1.3 mg protein) in Tris-Gly buffer (25 mM Tris–HCl and 192 mM Gly pH 8.3) and concentrated to a volume of 100 µL by centrifugation at 8,000 g for 20 min at 4 °C with a Millipore filter (100,000 Da). This highly purified fraction containing PEPC, in which proteolytic activity was absent, was named “electro-eluted PEPC” (Fig. 2b, lane 4).

### Protein quantification

We determined total protein amounts with Bradford’s (1976) method using bovine serum albumin (BSA) as the standard.

### Statistics

All data in this report were obtained from at least three independent experiments. Values are means ± SD (*n* ≥ 3).

## Results

### Proteolysis of C_4_-PEPC from sorghum leaves in the presence of pC19

**Fig. 1:**
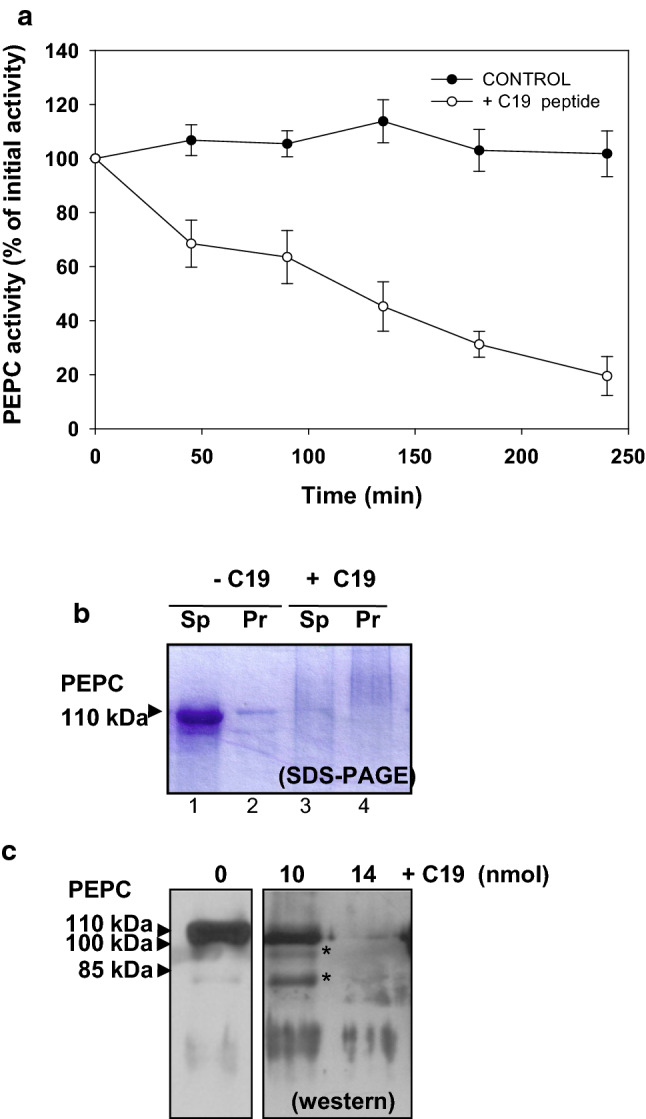
Effect of pC19 on PEPC activity/amount. PEPC protease activity was followed in 50 µL of incubation medium containing 0.3 U sp-PEPC, ± 60 nmol of pC19 at 30 ºC. **a** Time course of PEPC activity during the incubation assay (5 µL aliquot, pH 8 and 2.5 mM of PEP) in the absence or presence of pC19 (100% corresponding to 0.3 U PEPC and value represent the mean ± SD of three independent experiments). **b** SDS–PAGE analysis of PEPC amounts: after 3 h of incubation, samples were centrifuged (12.000 g, 10 min) and supernatant (Sp) and precipitate (Pr) were analyzed by SDS–PAGE and proteins were stained with Coomassie blue. **c** Pattern of proteolysis of PEPC by proteases in sp-PEPC in the presence of low amount of pC19. Sp-PEPC (0.01 U) was incubated 1 h, at 30 ºC in the absence (PEPC) or presence of 10 or 14 nmol of pC19 (+ C19). The samples were rapidly denatured to minimize proteolysis and analyzed by SDS–PAGE (10% of acrylamide) and immunoblotting with PEPC-IgGs

A marked decrease in PEPC activity was observed when sp-PEPC was incubated (2–4 h at 30 °C) in the presence of a synthetic, hydrophobic peptide that mimics the sequence of the last 19 amino acids at the C-terminal end of the enzyme pC19 (Fig. 1, + C19).

**Fig. 2:**
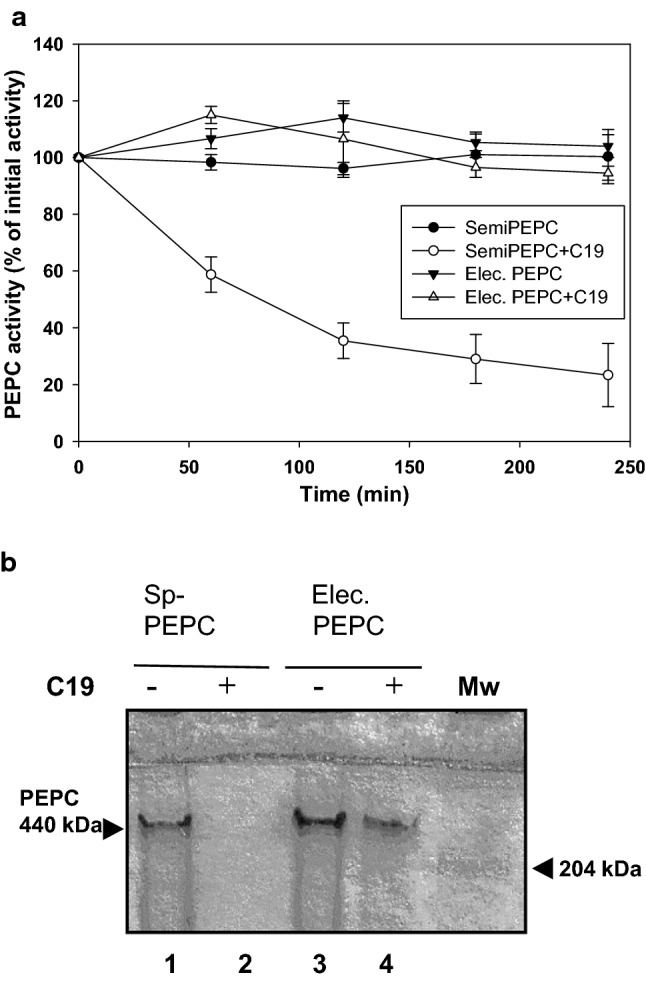
Native-PAGE separates PEPC from the protease and electro-eluted PEPC was not proteolysed in the presence of pC19**.** The sp-PEPC fraction was subjected to native PAGE and the PEPC protein band was electro-eluted. Aliquots containing 0.6 U of sp-PEPC or electro-eluted PEPC (Elec. PEPC) were incubated for 4 h, ± 60 nmol of pC19 (C19) in 50 µL of assay medium. **a** PEPC activity expressed as the percentage of the initial activity. Values represent the mean ± SD of 3 independent experiments). **b** Analysis by native-PAGE. MW, molecular weight markers

SDS–PAGE analysis of the sp-PEPC fraction after incubation with pC19 revealed that the decrease in PEPC activity was due to a loss of the corresponding protein (Fig. 1b, lane 3), whereas the enzyme remained stable in the control (Fig. 1b, lane 1). We checked that this decrease was not due to insolubilization of the enzyme following incubation (Fig. 1b, lanes 2 and 4). Immunoblots of aliquots incubated at low concentrations of pC19 indicated that PEPC was completely degraded after 3 h of incubation at 30 °C in the presence of 14 nmol pC19 (Fig. 1c). Incubation with a lower amount of peptide (10 nmol) resulted in the accumulation of PEPC fragments. These fragments were approximately 100 and 85 kDa (Fig. 1c, *). These results led us to believe that the loss of PEPC activity was due to the proteolysis of PEPC in the presence of pC19. To confirm this, we subjected sp-PEPC to an additional purification step in native PAGE (7% acrylamide) to obtain pure PEPC without proteolytic activity. First PEPC was localized with an in-gel activity assay, then the corresponding region was cut out and the PEPC recovered by electro-elution. In this case, incubation of the electro-eluted, highly purified PEPC with pC19 did not affect the enzyme activity (Fig. 2a) or the integrity of the protein (Fig. 2b, lane 4). This indicated that the proteases in sp-PEPC had indeed been separated from PEPC on native PAGE. In addition, the results confirmed that the loss of PEPC activity in the presence of pC19 was due to a proteolytic process. It is important to point out that in the absence of proteolytic activity, pC19 did not affect PEPC activity (Fig. 2a). Moreover, no higher molecular complexes were observed in the presence of the aforementioned peptide (Fig. 2b).

**Fig. 3:**
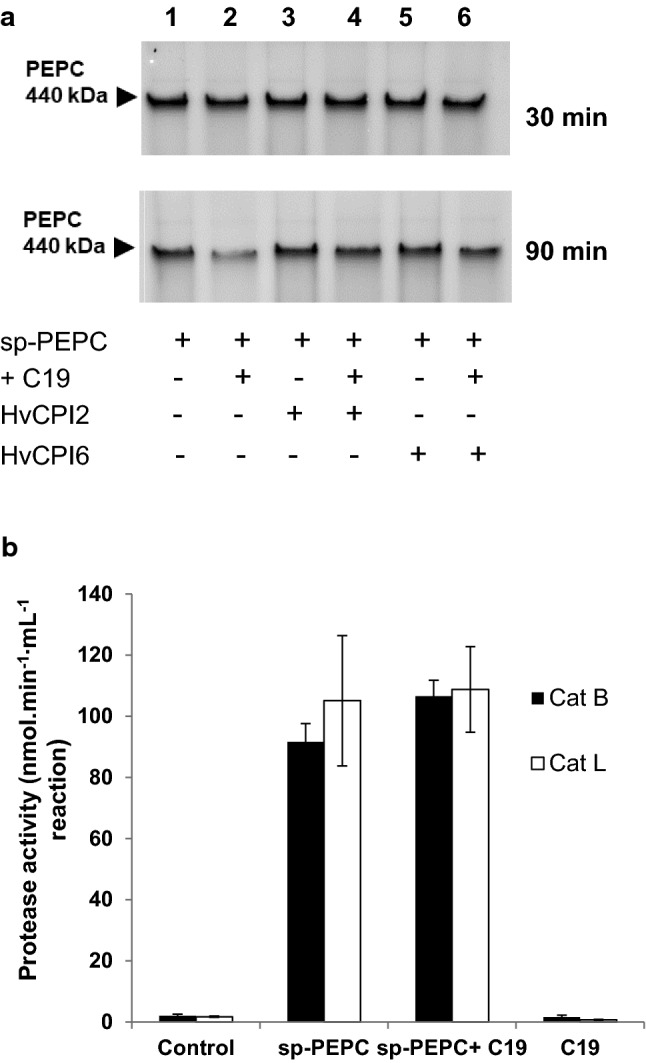
Cystatin inhibits the protease activity in sp-PEPC in the presence of pC19. **a** An aliquot of 0.2 U of sp-PEPC was incubated alone or in the presence of pC19 (+ C19; 60 nmol) and 1 µM of recombinant purified barley cystatins, *Hordeum vulgate* cystatin protein HvCPI2 and HvCPI6, in a final volume of 50µL containing 100 mM of citrate buffer at pH 6. The incubation was carried at 37^◦^C for 30 or 90 min. Finally, samples were subjected to native PAGE (7% acrylamide) and stained with Coomassie Blue. The bands intensity was quantified using the software PCBAS 2.0 and expressed as % respect to the control. **b** The protease activity present in sp-PEPC is independent of the presence of pC19 (+ C19). Cathepsin B-like (Cat-B) and L-like (Cat-L) protease activities were assayed as described in Materials and methods using Z-RR-AMC and Z-FR-AMC specific fluorescent substrates respectively, and a citrate buffer, pH 6. The reaction was incubated at 37 °C for 24 h. The system was calibrated with known amount of AMC in a standard reaction mixture. Control, used to account the spontaneous breakdown of substrates; sp-PEPC and sp-PEPC + C19 are sp-PEPC fraction in the absence or presence of pC19; C19, pC19 alone. Values represent the mean ± SD of three independent experiments

Previous results by our group had demonstrated that the main proteolytic activity present in sp-PEPC was cathepsin B and L from the cysteine protease superfamily (Gandullo et al. 2019). Cathepsin proteases are specifically inhibited by cystatins (Yamada et al. 2000; Martínez and Díaz 2008). To determine whether cathepsin proteases were responsible for the in vitro degradation of PEPC in the presence of pC19, we performed a proteolytic assay of PEPC in the presence and absence of the cystatins HvCPI2 and HvCPI6. As shown in Fig. 3, PEPC was partially degraded after 90 min of incubation with pC19 (lane 2), whereas proteolysis decreased in the presence of HvCPI2 and HvCPI6 (lanes 4 and 6).

We also assayed proteolytic activity with the commercial fluorescent substrates Z-RR-AMC and Z-FR-AMC for Cat-B and Cat-L proteases, respectively, to determine whether the effect of pC19 was on the protease activity or on PEPC. Figure 3b shows the degradation of these fluorescent substrates regardless of the presence of pC19. This proves that PEPC is the target of pC19.

### Thr present in pC19 is an important requirement for pC19-PEPC interaction

**Fig. 4:**
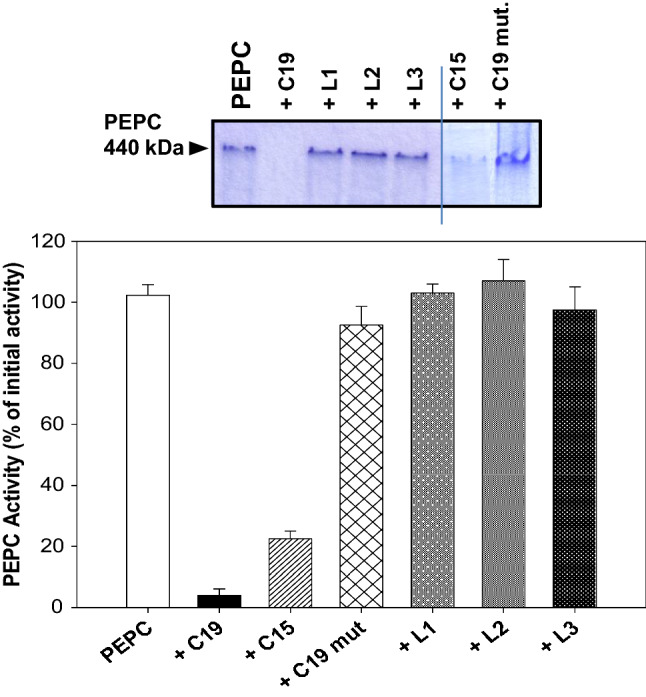
Specificity of pC19 promoting PEPC proteolysis. Experiments were performed as described in the legend of Fig. 1 using 60 nmol for peptides L1, L2, L3, pC19 (+ C19), pC15 (+ C15) or pC19-mut (+ C19 mut). After 3 h of incubation at 30 ºC, PEPC activity was measured at pH 8 and 2.5 mM of PEP. The activity was expressed as the percentage of the initial activity (graphic). At the end of the incubation time aliquots (0.15 U PEPC) from the different samples were analyzed by native PAGE (7% acrylamide). Protein in gel was stained with Coomassie Blue. Values represent the mean ± SD of three independent experiments. Peptides are described in Materials and methods

The C-terminal QNTG domain is indispensable for maximal catalytic activity but not for the homotetramer formation of sorghum C_4_-PEPC (Dong et al. 1999). To determine whether this conserved domain is essential to promote PEPC proteolysis, we assayed a truncated pC19 lacking the last four amino acids QNTG (pC15) and found it to be as effective as pC19 at triggering PEPC proteolysis (Fig. 4, + C15). Other synthetic peptides—L1 and the hydrophobic peptides L2 and L3, designed from computer-based modeling of the sorghum C_4_-PEPC (Kai et al. 1999; Matsumura et al. 2002), corresponding to solvent-exposed protein loops were tested but all failed to promote proteolysis of the enzyme (Fig. 4, L1, L2, L3). These data suggest that hydrophobicity alone cannot account for the positive effect of pC19 on PEPC proteolysis. This view is well supported by the fact that a mutated pC19 with an increased hydrophobic index (Thr944, Thr948, and Thr959 replaced by Tyr residues [(Y)942ED**Y**_**944**_LIL**Y**_**948**_MKGIAA GMQN**Y**_**959**_G960]) lost its ability to promote PEPC proteolysis (Fig. 4, + C19 mut). Thus, our data suggest that pC19 is specifically required to enhance PEPC proteolysis and that Thr944 and Thr948 are important requirements for this interaction. Thus, direct interaction between the C-term of a PEPC with another native PEPC subunit could take place. The feasibility of this mechanism in vivo remains to be investigated. In the meantime, we have used pC19 as a tool to promote the proteolysis of PEPC and to study the interplay among the oligomerization state, metabolites, or the phosphorylation state of the enzyme in its proteolysis.

### Monomeric and dimeric PEPC are more sensitive to proteolysis

**Fig. 5:**
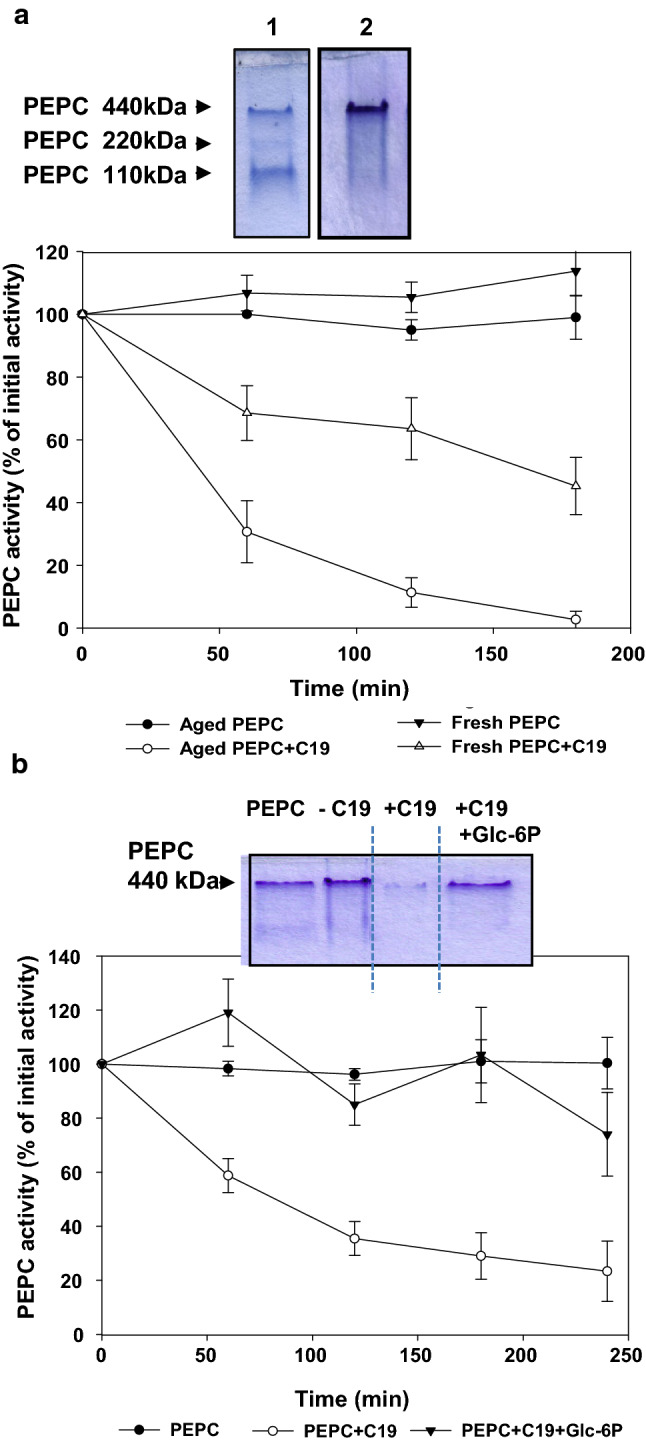
The oligomerization state of PEPC and Glc-6P affect the sensitivity to proteolysis in the presence of pC19. **a** Coomassie stained PEPC in native PAGE (7% acrylamide). Lane 1 corresponds to a preparation of 2 month old of sp-PEPC conserved at − 20 ºC (aging PEPC) and lane 2 contains freshly prepared sp-PEPC. Both lanes were loaded with 6 μg protein. The graphic represents a time course of PEPC activity in the incubation assay in the absence or presence of pC19 (+ C19). 100% corresponds to 0.3 U PEPC and data are means of three independent experiments. **b** PEPC amount and activity after incubation with pC19 (+ C19), or pC19 and 5 mM Glc-6P (+ C19 + Glc-6P). A similar time course as described above was done and samples (0.15 U PEPC) were analyzed by native PAGE. Values represent the mean ± SD of 3 independent experiments)

Aging the sp-PEPC fraction (2–3 months at −20 °C) led to increased amounts of dimeric and monomeric enzyme species (Fig. 5a, lane 1 vs. lane 2). This was easily detected because PEPC was the dominant protein in the sp-PEPC preparation (lane 2). In the case of aging PEPC, proteolysis in the presence of pC19 was strongly enhanced compared to the freshly prepared enzyme (Fig. 5b, graphic). This suggests that the tetrameric form is the most stable conformation in the presence of the cathepsin proteases described to be present in sp-PEPC (Gandullo et al. 2019).

### Gluc-6P prevents the proteolysis of PEPC

We then determined whether regulatory metabolites of PEPC affect the proteolysis of the enzyme in the presence of pC19. It is interesting that the loss of PEPC activity in the presence of pC19 was prevented by the allosteric activator Glc-6P (Fig. 5b, graphic). Glc-6P positively regulates PEPC by increasing its activity and preventing the subunit dissociation of the enzyme (Chollet et al. 1996). Recently, we demonstrated that Glc-6P not only prevents the exposure of the C-term of the native PEPC but returns the exposed sequence to the embedded native conformation (Gandullo et al. 2019). This indicates that Glc-6P is not only a positive effector of the enzyme’s activity as largely described in the literature (Chollet et al. 1996) but also a protector of its integrity.

### Phosphorylated PEPC is less sensitive to proteolysis

**Fig. 6:**
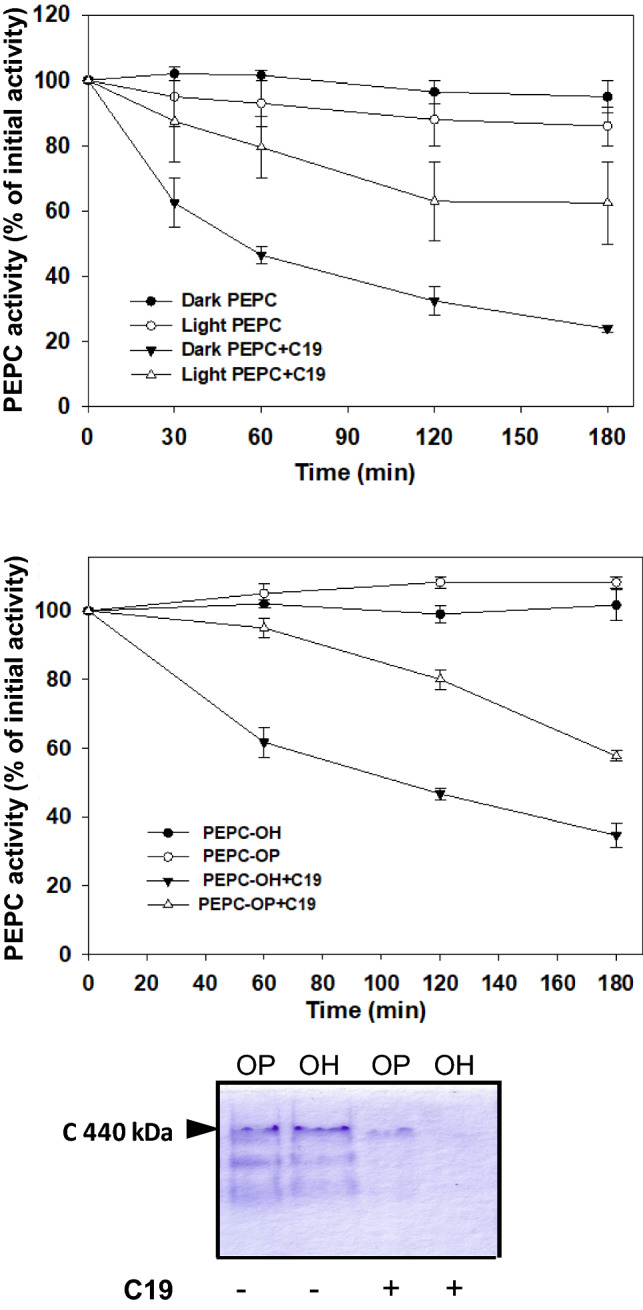
Phosphorylated PEPC is less sensitive to proteolysis than dephosphorylated PEPC. **a** PEPC protease activity was followed during 4 h, at 30 ºC, in crude extract from illuminated (Ligh PEPC) or darkened (Dark PEPC) sorghum leaves containing 0.3 U PEPC and in the absence or presence of 60 nmol of the pC19 (+ C19). At the indicated time, PEPC activity was determined at pH 8 and 2.5 mM of PEP. **b** The same as in **a** but using sp-PEPC fraction previously phosphorylated or not with the catalytic subunit of the cAMP-dependent protein kinase (PKA). **c** The same as that in **b** but the analysis of the protein was performed by native PAGE (7% acrylamide). The IC_50_ values for L-malate of PEPC in crude extracts or in vitro phosphorylated PEPC are shown in the graphics **a** and **b**, respectively, and was determined at pH 7.3 and 2.5 mM of PEP. Values represent the mean ± SD of 3 independent experiments

In C_4_ plants, C_4_-PEPC is subjected to diel post-translational regulation that alters its functional and regulatory properties (Chollet 1996; Echevarría and Vidal 2003). This occurs when a small molecular mass (32 kDa) phosphoenolpyruvate carboxylase kinase (PEPCk) phosphorylates the Ser of the enzyme’s N-terminal domain (E/DR/KxxSIDAQL/MR; Jiao et al. 1991). When performed at suboptimal pH (7.3) and 2.5 mM PEP, it has been established that the IC_50_ for L-malate (the concentration of L-malate that inhibits PEPC activity by 50%) reflects the phosphorylated state of PEPC. Values around 1.2 and 0.4 mM IC_50_ represent phosphorylated and dephosphorylated enzymes, respectively (McNaughton et al. 1989; Echevarría et al. 1990, 1994). Taking advantage of pC19 to promote the proteolysis of PEPC, we explored how the phosphorylation state of the enzyme impacts on the sensitivity of the protein to proteolysis. This was done with crude extracts from illuminated (phosphorylated PEPC) or darkened (dephosphorylated PEPC) sorghum leaves. Furthermore, we phosphorylated PEPC in semi-purified fraction in vitro using the catalytic subunit of protein kinase (PKA) (an efficient PEPCk, although one thus far not found in plants; Terada et al. 1990). Previously to the addition of pC19, the phosphorylation state of PEPC was confirmed by L-malate test (IC_50_) (IC_50_ values shown in Fig. 6a, b). As shown in Fig. 6, the phosphorylated PEPC was less sensitive to proteolysis than the dephosphorylated form. This was demonstrated by the loss of PEPC activity in the presence of pC19 and by the amount of dephosphorylated and phosphorylated sp-PEPC in PAGE after incubation in the presence of pC19 (Fig. 6c).

**Fig. 7:**
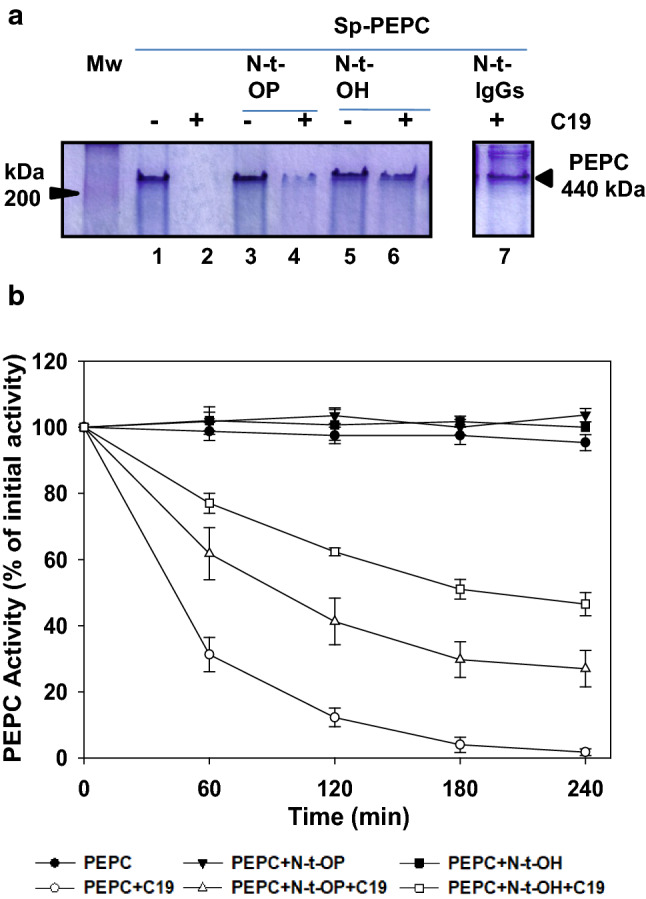
N-terminal-(N-t) dephospho-peptide (N-t-OH) is a better inhibitor of PEPC proteolysis in the presence of pC19 than the N-t-phospho-peptide (N-t-OP). **a** The N-t-peptides (N-t peptide; 60 nmol) were added (lanes, 3, 4, 5 and 6) or not (lanes 1, 2 and 7) to the proteolytic assay with sp-PEPC (0.3 Unit), in the presence (lanes 2, 4, 6 and 7) or absence (lanes 1, 3, 5) of 60 nmol of pC19 (+ C19). Lanes 3 and 4, phosphopeptide (N-t-OP); lanes 5 and 6 dephospho-peptide (N-t-OH). Lane 7 is similar to lane 2 but in the presence of specific antibodies against peptide N-t-OH (N-t-IgGs). After 3 h incubation at 30 ºC samples were analyzed by native PAGE (7% acrylamide). Proteins were stained with Coomassie blue. MW, molecular weight. **b** Time course of PEPC activity in the presence or absence of N-t-peptide and pC19 (+ C19). The proteolytic assay was performed in standard condition in the presence of 60 nmol of each peptides (N-t-OH) or (N-t-OP). 100% activity corresponds to 0.3 U PEPC. Values represent the mean ± SD of three independent experiments

Proteolysis of the N-terminal end during the purification of PEPC has been extensively reported (McNaughton et al. 1989; Baur et al. 1992; Crowley et al. 2005). In addition, C_3_-PEPC (RcPPC3) from castor oil seeds exists in vivo as a proteolyzed N-terminal subunit (Uhrig et al. 2008; O’Leary et al. 2011). Similarly, we recently demonstrated that the loss of the N-terminal end of PEPC is an early event in proteolysis conducted by cathepsin proteases present in sp-PEPC in the presence of PA (Gandullo et al. 2019). Here we show that the proteolysis of PEPC was blocked in the presence of N-t-IgGs antibodies (produced against the N-t-peptide) in the proteolytic assay (Fig. 7, lane 7 vs. lane 2). In addition, PEPC proteolysis in the presence of pC19 was inhibited by the phosphorylated N-terminal peptide (N-t-OP; Fig. 7a, lane 4). However, this effect was much more pronounced when a N-terminal dephosphorylated peptide (N-t-OH) was used (Fig. 7a, lane 6), which suggests that N-t-OH is a better inhibitor of the proteases in this sp-PEPC reaction than N-t-OP.

Collectively, these results establish that phosphorylated PEPC is less suitable for degradation by cathepsin proteases present in sp-PEPC than the dephosphorylated enzyme. This is the first report to show that the regulatory phosphorylation of C_4_-PEPC is a mechanism for preserving the integrity of the enzyme.

## Discussion

The C-term of PEPC is a highly conserved sequence whose last 19 amino acids are present in all plant-type PEPCs (PTPCs) sequenced to date. It has been described as a hydrophobic alpha-helix sequence embedded in the subunit (Kai et al. 2003) in the active and stable conformation of the enzyme (Álvarez et al. 2003; Kay et al. 2003; Gandullo et al. 2019). The crystalline structure of maize PEPC shows that this sequence oscillates between the active (R) and non-active (T) state of the protein. The R state is stabilized via interaction with the terminal glycine of the C-term, which suggests the importance of the C-term in both catalytic activity and allosteric regulation (Izui et al. 2004). Along the same lines, using specific C-term IgGs (pC19-IgGs) and immunoprecipitation assay, we have demonstrated that the C-term of PEPC can be in two conformational states in vitro, embedded or exposed, being the embedded conformation the most abundant (Álvarez et al. 2003) and active conformation of the enzyme (Gandullo et al. 2019). This was investigated further to show that in vitro PA and anionic phospholipids trigger the exposed C-term conformation, this change being correlated to its proteolysis by cathepsin proteases of the leaf extract (Monreal et al. 2010; Gandullo et al. 2019). The C-term has been also described as an important requirement for the stability of the enzyme since a mutated PEPC lacking this sequence is degraded in *E. coli* (Dong et al. 1999). Therefore, it was tempting to examine whether the C-term is a major player in the largely unknown mechanism of PEPC proteolysis/stability. As the C-term is normally embedded in the subunit, pC19 represents an efficient tool to study this possible interaction. Very unexpectedly, we found that when pC19 was added to sp-PEPC from darkened sorghum leaves, PEPC activity was rapidly lost. This was due to proteolytic degradation of the enzyme by cathepsin proteases present in the sp-PEPC fraction. In addition, in the absence of these proteases, the activity and integrity of the enzyme were not affected (Fig. 2, lane 4). The last four amino acids QNTG at the end of the pC19 sequence are not a necessary requirement for interaction between PEPC and pC19, as a pC15 peptide lacking these four amino acids was equally effective as pC19 at promoting the proteolysis of the enzyme (Fig. 4). However, the Thr944 and Thr948 residues are important requirements, as pC19 in which these Thr had been changed by Tyr showed a complete loss of efficiency. The interaction between pC19 and PEPC seems to be specific, as other peptides containing sequences of PEPC (L1, L2, and L3) were ineffective promoting the proteolysis (Fig. 4). Thus, one main requirement for pC19 to be active is hydrophobicity, but specificity of the amino acid sequence is also required.

It is thus reasonable to assume that pC19, PEPC fragment in which a C-term peptide could be found or the exposed C-term of a PEPC subunit could interact in vivo with other native PEPC to promote the exposure of new cleavage sites and to increase its sensitivity to proteolysis. In a previous study, we reported novel results supporting the viability of this hypothesis (Gandullo et al. 2019). For example, PA increases in response to different stresses (Munnik 2001), including salinity in sorghum plants (Monreal et al. 2013). PA and other anionic phospholipids change C_4_-PEPC to a C-term-exposed conformation, which greatly increases the susceptibility of the enzyme to proteolyzation (Gandullo et al. 2019). In contrast, Glc-6P maintains the enzyme in a conformation with an unexposed C-term (Gandullo et al. 2019) and preserves the stability of the enzyme (Fig. 5b). These data demonstrate that there are, in vivo, potential regulators of the exposition of the C-term of PEPC making possible to increase or decrease the sensitivity of the enzyme to proteolysis, and PEPC fragments with the C-term may to contribute to the autocatalysis of the processes. However, in this work we used a high ratio of pC19 to the PEPC protein, which suggests that the in vivo conditions in which this interaction can take place (oligomeric state of the enzyme, the real size of the peptides, or the presence of metabolites) may be essential for the interaction.

We also studied how the conformation of PEPC, regulatory metabolites, or the phosphorylation of PEPC affect the stability of the enzyme. The results presented here show that proteolysis in the presence of pC19 can be modulated by the oligomeric state of PEPC, with the tetrameric form the most stable (Fig. 5a). These results are in good agreement with a report by Weding and Black (1987) showing that the sensitivity of a CAM (Crassulacean acid metabolism) PEPC to proteases was clearly related to its oligomeric state. In addition, we have proven here that Glc-6P decreases the degradation of PEPC in the presence of pC19 (Fig. 5b). As described previously by our group, Glc-6P prevents the exposure of the C-term and returns the exposed C-term conformation to the embedded state (Gandullo et al. 2019). This result suggests that an exposed or embedded C-term is key to regulating the susceptibility of the enzyme to proteolysis and describes a new role for Glc-6P not only as a positive effector (Ghollet et al. 1996) but also as a stabilizer of the integrity of the enzyme (Gandullo et al. 2019; and Fig. 5b in this work).

To date, the role assigned to the reversible phosphorylation of PEPC is to affect the catalytic and regulatory properties of the enzyme (Duff et al. 1995; Nimmo 2003). However, although this has been largely demonstrated, the lack of PEPC kinases (PEPCks) in a *Flaveria bidentis* mutant had no effect on photosynthetic rates (Furumoto et al. 2007). Additionally, it is difficult to explain why a modified form of C_4_- or CAM-PEPC whose N-terminal region is truncated by proteolysis shows marked desensitization to Asp or L-malate (Chollet et al. 1996; Izui et al. 2003). Furthermore, a C_3_-PEPC from castor oil seed (RcPPC3) exists in vivo as a proteolyzed N-terminal subunit (Uhrig et al. 2008; O’Leary et al. 2011). This calls into question the unique role assigned to date to the phosphorylation of C_4_-PEPC in C_4_ photosynthesis and prompted us to look for alternative functions (e.g., regulation of the stability of the enzyme). Results of this work using pC19 prove that phosphorylation of PEPC decreases the susceptibility of the enzyme to proteolysis.

In vivo phosphorylated PEPC from illuminated leaves showed a slowed kinetic degradation in the presence of pC19 compared to the dephosphorylated enzyme. Moreover, in vitro phosphorylated PEPC was less susceptible to degradation than the dephosphorylated form (Fig. 6). It is interesting that the addition of N-t-IgGs raised against the N-terminus of sorghum C_4_-PEPC efficiently decreased the proteolysis of the enzyme (Fig. 7a, lane 7). Saturation of dephospho-PEPC with specific N-t-IgGs antibodies promotes a marked alteration of C_4_-PEPC functional and regulatory properties mimicking a phosphorylated enzyme (Pacquit et al. 1995). These antibodies may also block access to some essential cleavage sites and hinder proteolysis. It is curious that the addition of dephospho- or phospho-N-terminal peptides decreased the proteolysis of PEPC in the presence of pC19. However, Fig. 7 indicates that the dephosphorylated peptide was a better inhibitor of PEPC proteolysis than the phosphorylated peptide. Collectively, these results describe a new role for the reversible phosphorylation of PEPC. PEPC phosphorylation affects not only the regulatory and catalytic properties of PEPC (Echevarría et al. 1994) but also the stability of the enzyme. In line with these results, C_4_-PEPC from salt-treated sorghum plants was highly phosphorylated (Echevarría et al. 2001), and the enzyme was much more stable against degradation than those in control conditions (García-Mauriño et al. 2003; Monreal et al. 2007). Conversely, phosphorylation of PEPC from guard cells of *Vicia faba*, a C_3_-PEPC isoenzyme, participates in the enzyme’s degradation (Klockenbring et al. 1998).

In this work, we demonstrate that dephosphorylated PEPC is a better substrate than phosphorylated PEPC for the cathepsin proteases present in sp-PEPC. All together, these data allow us to suggest a model in which the C-term of PEPC may be exposed or embedded; molecules in the first group would not be phosphorylated (PEPCk would be inhibited by the exposed C-term; Álvarez et al. 2003) and would ideally be sent for proteolysis, whereas those in the second group would be phosphorylated, thus increasing the stability of the enzyme. In this model, Glc-6P and PA trigger each conformation, with Glc-6P acting as a protector and PA acting as an inducer of enzyme degradation. It is interesting that the presence of Glc-6P and PEPC phosphorylation are related to the active phase of CO_2_ fixation in C_4_ photosynthesis (Nimmo et al. 2003), whereas increased PA in the cell is related to stress processes (Testerink and Munnik 2005).

## *Author contribution statement *

CE conceived the research hypothesis. CE, JG, RA, and JV designed the study and conducted the research. ABF and JAM co-supervised the research. ID provided fluorescent substrates of proteases and cystatin for the protease characterization experiments, which were performed in her laboratory by JG. CE wrote the manuscript.

## Supplementary Information

Below is the link to the electronic supplementary material.

Supplementary file1 (DOCX 1381 KB)

## Data Availability

All data generated or analyzed during this study are included in this published article.

## Abbreviations

C-term: C-terminal 19 amino acids of sorghum C_4_-PEPC
pC19: Conserved 19 amino acid synthetic peptide from the carboxy terminus of sorghum C_4_-PEPC
Glc-6P: Glucose 6-phosphate
PA: Phosphatidic acid
PEP: Phosphoenolpyruvate
PEPC: Phosphoenolpyruvate carboxylase
sp-PEPC: Semi-purified PEPC fraction with protease activity

## References

[CR1] Álvarez R, García-Mauriño S, Feria AB, Vidal J, Echevarría C (2003). A conserved 19 amino acids synthetic peptide from the carboxy terminus of phosphoenolpyruvate carboxylase inhibits the in vitro phosphorylation of the enzyme by the calcium-independent phosphoenolpyruvate carboxylase kinase. Plant Physiol.

[CR2] Arias-Baldrich C, de la Osa C, Bosch N, Ruiz-Ballesta I, Monreal JA, García-Mauriño S (2017). Enzymatic activity, gene expression and posttranslational modifications of photosynthetic and non-photosynthetic phosphoenolpyruvate carboxylase in ammonium-stressed sorghum plants. J Plant Physiol.

[CR3] Baena G, Feria AB, Echevarría C, Monreal JA, García-Mauriño S (2017). Salinity promotes opposite patterns of carbonylation and nytrosilation of C_4_ phosphoenolpyruvate carboxylase in sorghum leaves. Planta.

[CR4] Baena G, Feria AB, Hernández-Huertas L, Gandullo J, Echevarría C, Monreal JA, García-Mauriño S (2021). Genetic and pharmacological inhibition of autophagy increases the monoubiquitination of non-photosynthetic phosphoenolpyruvate carboxylase. Plants.

[CR5] Baur B, Dietz KJ, Winter K (1992). Regulatory protein phosphorylation of phosphoenolpyruvate carboxylase in the facultative crassulacean-acid-metabolism plant *Mesembryanthemun crystallinum* L. Eur J Biochem.

[CR6] Bradford MM (1976). A rapid and sensitive method for the quantitation of microgram quantities of protein utilizing the principle of protein-dye binding. Anal Biochem.

[CR7] Carrillo L, Martínez M, Ramessar K, Cambra I, Castañeda P, Ortego P, Díaz I (2011). Expression of barley cystatine gene in maize enhances resistance against phytophagous mites by altering their cystein proteases. Plant Cell Rep.

[CR8] Chollet R, Vidal J, O’Leary MH (1996). Phosphoenolpyruvate carboxylase: a ubiquitous, highly regulated enzyme in plants. Annu Rev Plant Physiol Plant Mol Biol.

[CR9] Crowley V, Gennidakis K, Plaxton WC (2005). In vitro proteolysis of phosphoenolpyruvate carboxylase from developing castor oil seeds by an endogenous thiol endopeptidase. Plant Cell Physiol.

[CR10] Dong L, Patil S, Condon S, Hass E, Chollet R (1999). The conserved C-terminal tetrapeptide of sorghum C_4_ phosphoenolpyruvate carboxylase is indispensable for maximal catalític activity, but not for homotetramer formation. Arch Biochem Biophys.

[CR11] Duff SMG, Andreo CS, Pacquit V, Lepiniec L, Sarath G, Condon SA, Vidal J, Chollet GP (1995). Kinetic analysis of the non-phosphorylated, in vitro phosphorylated, and phosphorylation-site-mutant (Asp8) forms of intact recombinant C_4_ phosphoenolpyruvate carboxylase from sorghum leaves. Eur J Biochem.

[CR12] Echevarría C, Vidal J (2003). The unique phosphoenolpyruvate carboxylase kinase. Plant Physiol Biochem.

[CR13] Echevarría C, Vidal J, Jiao JA, Chollet R (1990). Reversible light activation of the phosphoenolpyruvate carboxylase protein-serine kinase in maize leaves. FEBS Lett.

[CR14] Echevarría C, Pacquit V, Bakrim N, Osuna L, Delgado B, Arrio-Dupont M, Vidal J (1994). The effect of pH on the covalent and metabolic control of C_4_ phospho*enol*pyruvate carboxylase from sorghum leaf. Arch Biochem Biophysics.

[CR15] Echevarría C, García-Mauriño S, Álvarez R, Soler A, Vidal J (2001). Salt stress increases the Ca^2+^- independent phospho*enol*pyruvate carboxylase kinase activity in sorghum leaves. Planta.

[CR16] Furumoto T, Izui K, Quinn V, Furbank RT, Von Caemmerer S (2007). Phosphorylation of phosphoenolpyruvate carboxylase is not essential for high photosynthetic rates in the C4 species *Flaveria bidentis*. Plant Physiol.

[CR17] Gandullo J, Monreal JA, Álvarez R, Díaz I, García-Mauriño S, Echevarría C (2019). Anionic phospholipids induce conformational changes in phosphoenolpyruvate carboxylase to increase sensitivity to cathepsin proteases. Front Plant Sci.

[CR18] García-Mauriño S, Monreal JA, Álvarez R, Vidal J, Echevarría C (2003). Characterization of salt stress-enhanced phosphoenolpyruvate carboxylase kinase activity in leaves of *Sorghum vulgare*: independence from osmotic stress, involvement of ion toxicity and significance of dark phosphorylation. Planta.

[CR19] Gehrig HH, Valentina H, Kluge M (1998). Towards a better knowledge of the molecular evolution of phosphoenolpyruvate carboxylase by comparison of partial cDNA sequences. J Mol Evol.

[CR20] Grisvard J, Keryer E, Takvorian A, Dever LV, Lea PJ, Vidal J (1998). A splice site mutation gives rise to a mutant of the C_4_ plant *Amaranthus edulis* deficient in phosphoenolpyruvate carboxylase activity. Gene.

[CR21] Hewitt EJ (1966). The composition of the micronutrient solution. In: sand and water culture methods in the study of plants nutrition.

[CR22] Izui K, Tsuchida Y, Agetsuma M, Ohshima K, Furumoto T (2003). Enzymatic characterization of the recombinant phosphoenolpyruvate carboxylase kinase (PEPCPK) from a C4 plant, *Flaveria trinervia*: Possible redox regulation. Presented at Annu Meet Am Soc Plant Biol Honolulu.

[CR23] Izui K, Matsumura H, Furumoto T, Kai Y (2004). Phosphoenolpyruvate carboxylase: a new era of structural biology. Annu Rev Plant Biol.

[CR24] Jiao JA, Vidal J, Echevarría C, Chollet R (1991). In vivo regulatory phosphorylation site in C_4_-leaf phosphoenolpyruvate carboxylase from maize and sorghum. Plant Physiol.

[CR25] Kai Y, Matsumura H, Inoue T, Terada K, Nagara Y, Yoshinaga T, Kihara T, Tsumura K, Izui K (1999). Tree-dimensional structure of phosphoenolpyruvate carboxylase: a proposed mechanism for allosteric inhibition. Proc Natl Acad Sci USA.

[CR26] Kai Y, Matsumura H, Izui K (2003). Phosphoenolpyruvate carboxylase: three dimensional structure and molecular mechanisms. Arch Biochem Biophys.

[CR27] Klockinbring T, Meinhard M, Schnabl H (1998). The stomatal phosphoenolpyruvate carboxylase a potential target for selective proteolysis during stomatal closure?. J Plant Physiol.

[CR28] Laemmli UK (1970). Cleavage of structural proteins during the assembly of the head of bacteriophage T4. Nature.

[CR29] Lepiniec L, Keryer E, Philippe H, Gadal P, Cretin C (1993). *Sorghum* phosphoenolpyruvate carboxylase gene family: structure, function and molecular evolution. Plant Mol Biol.

[CR30] Martínez M, Díaz I (2008). The origin and evolution of plant cystatins and their target cysteine proteinase indicate a complex functional relationship. BMC Evol Biol.

[CR31] Matsumura H, Xie Y, Shirakata S, Inoue T, Yoshinaga T, Ueno Y, Izui K, Kai Y (2002). Cristal structure of C_4_ form maize and quaternary complex of *E. coli* phosphoenolpyruvate carboxylase. Structure.

[CR32] McNaughton GAL, Fewson CA, Wilkins MB, Nimmo HG (1989). Purification, oligomerization state and malate sensitivity of maize leaf phosphoenolpyruvate carboxylase. Biochem J.

[CR33] McNaughton GA, Fewson CA, Wilkins MB, Nimmo HG (1989). Purification, oligomerization state and malate sensitivity of maize leaf phosphoenolpyruvate carbaxylase. Biochem J.

[CR34] Monreal JA, Feria AB, Vinardell JM, Vidal J, Echevarría C, García-Mauriño S (2007). ABA modulates the degradation of phosphoenolpyruvate carboxylase kinase in sorghum leaves. FEBS Lett.

[CR35] Monreal JA, McLoughlin F, Echevarría C, García-Mauriño S, Testerink C (2010). Phosphoenolpyruvate carboxylase from C_4_ leaves is selectively targeted for inhibition by anionic phospholipids. Plant Physiol.

[CR36] Monreal JA, Arias-Baldrich C, Pérez-Montaño F, Gandullo J, Echevarría C, García-Mauriño S (2013). Factors involved in the rise of phosphoenolpyruvate carboxylase-kinase activity caused by salinity in sorghum leaves. Planta.

[CR37] Munnik T (2001). Phosphatidic acid: an emerging plant lipid second messenger. Trends Plant Sci.

[CR38] Nimmo HG (2003). Control of phosphorylation of phosphoenolpyruvate carboxylase in higher plants. Arch Biochem Biophys.

[CR39] O’Leary B, Park J, Plaxton WC (2011). The remarkable diversity of plant PEPC (phosphoenolpyruvate carboxylase): recent insights into the physiological function and post-translational controls of non-photosynthetic PEPC. Biochem J.

[CR40] Pacquit V, Giglioli N, Crétin C, Pierre JN, Vidal J, Echevarría C (1995). Regulatory phosphorylation of C4 phosphoenolpyruvate carboxylase from *Sorghum*: An immunological study using specific anti-phosphorylation site antibodies. Photosynth Res.

[CR41] Paterson AH, Bowers JE, Bruggmann R (2009). The *Sorghum bicolor* genome and the diversification of grasses. Nature.

[CR42] Plaxton WC (2019). Avoiding proteolysis during the extraction and purification of active plant enzymes. Plant Cell Phys.

[CR43] Ruíz-Ballesta I, Feria AB, Ni H, She YM, Plaxton WC, Echevarría C (2014). In vivo monoubiquitination of anaplerotic phosphoenolpyruvate carboxylase occurs at Lys624 in germinating sorghum sedes. J Exp Bot.

[CR44] Ruíz-Ballesta I, Baena G, Gandullo J, Wang L, She YM, Plaxton WC, Echevarría C (2016). New insights into the post-translational modification of multiple phosphoenolpyruvate carboxylase isoenzymes by phosphorylation and monoubiquitination during sorghum seed development and germination. J Exp Bot.

[CR45] Sabe H, Miwa T, Kodaki T, Izui K, Hiraga S, Katsuki H (1984). Molecular cloning of the phosphoenolpyruvate carboxylase gene, *ppc*, of *Escherichia coli*. Gene.

[CR46] Sánchez R, Cejudo FJ (2003). Identification and expression analysis of a gene encoding a bacterial–type phosphoenolpyruvate carboxylase from *Arabidopsis* and rice. Plant Physiol.

[CR47] Terada K, Kai T, Okuno S, Fujisawa H, Izui K (1990). Maize leaf phosphoenolpyruvate carboxylase: phosphorylation of Ser^15^ with mammalian cyclic AMP-dependent protein kinase diminishes sensitivity to inhibition by malate. FEBS Lett.

[CR48] Testerink C, Munnik T (2005). Phosphatidic acid: a multifunctional stress signaling lipid in plants. Trends Plant Sci.

[CR49] Uhrig RG, She YM, Leach CA, Plaxton WC (2008). Regulatory monoubiquitination of phosphoenolpyruvate carboxylase in germinating castor oil seeds. J Biol Chem.

[CR50] Wedding RT, Black MK (1987). Oligomerization and the sensitivity of phosphoenolpyruvate carboxylase to inactivation by proteinases. Plant Physiol.

[CR51] Yamada TY, Ohta H, Shinohara A, Iwamatsu A, Shimada H, Tsuchiya T, Masuda T, Takamiya K (2000). A cysteine protease from maize isolated in a complex with cystatin. Plant Cell Physiol.

