# Supplementary material for: A conserved C-terminal peptide of sorghum phosphoenolpyruvate carboxylase promotes its proteolysis, which is prevented by Glc-6P or the phosphorylation state of the enzyme
Source: Planta. 2021 Aug 5;254(3):43. doi: 10.1007/s00425-021-03692-3 (PMC8342391; doi:10.1007/s00425-021-03692-3)
Supplement: Supplementary file 1 — Supplementary file1 (DOCX 1381 KB) [file 425_2021_3692_MOESM1_ESM.docx]

**A conserved C-terminal peptide of sorghum phosphoenolpyruvate carboxylase promotes its proteolysis which is prevented by Glc-6P or the phosphorylation of the enzyme.**

**Supplemental Figures**

**Suppl. Fig S1** Whole gels used in Fig. 1c. The different samples, containing different amounts of pC19 were ran in two different gels, revealed at the same time with the same contrast. The lanes 0, 10 and 14 nmol C19 were selected for Fig. 1c


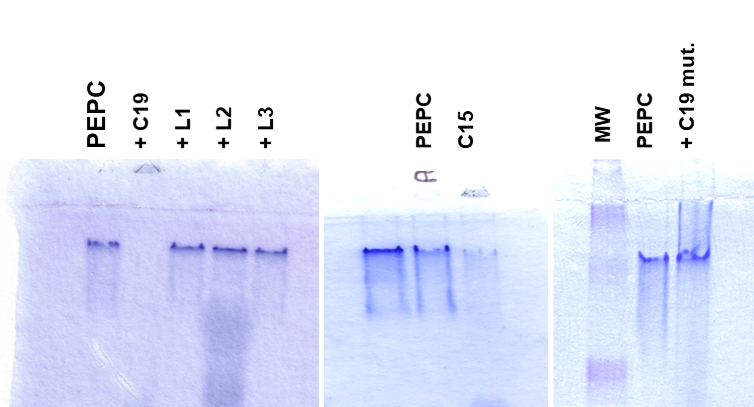


**Suppl. Fig. S2** Whole gels used in Fig 4 that include the corresponding control of each experiment. In the final Fig. 4 the different PEPCs control has been replaced for one PEPC control for a better understanding of the complete figure.


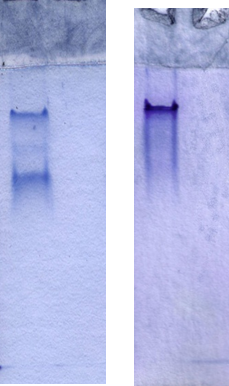


**Suppl. Fig. S3** Whole gels for dimeric PEPC (left), and tetrameric PEPC (righ) used in Fig. 5a


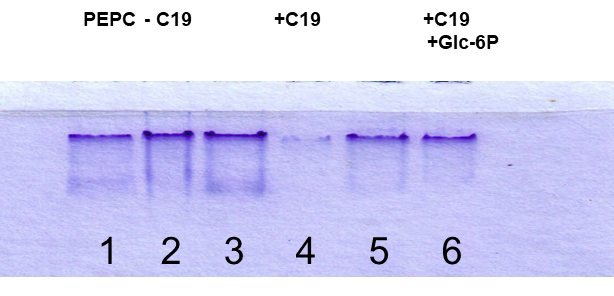


**Suppl. Fig. S4** Whole gels for Glc-6P effect. Lanes 1, 2, 4 and 6 were selected for Fig. 5b


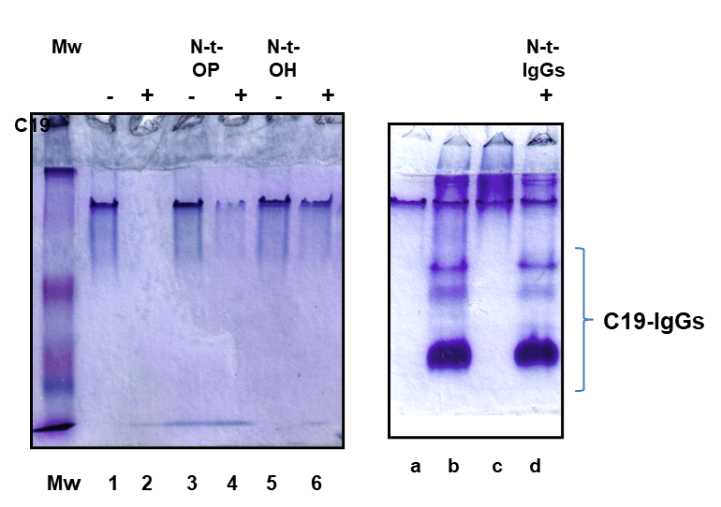


**Suppl. Fig. S5** Whole gels from Fig. 7. From the gel on the left all lanes were selected for Fig. 7. From the gel on the right, lane d was selected (that correspond to lane 7 in Fig. 7). The PEPC control loaded in lane 1 is similar to PEPC control in lane a therefore, PEPC control in lane 1 was selected for Fig 7
